# Cell morphology as a biological fingerprint of chondrocyte phenotype in control and inflammatory conditions

**DOI:** 10.3389/fimmu.2023.1102912

**Published:** 2023-02-13

**Authors:** Mischa Selig, Saman Azizi, Kathrin Walz, Jasmin C. Lauer, Bernd Rolauffs, Melanie L. Hart

**Affiliations:** ^1^ G.E.R.N. Research Center for Tissue Replacement, Regeneration & Neogenesis, Department of Orthopedics and Trauma Surgery, Faculty of Medicine, Medical Center—Albert-Ludwigs-University of Freiburg, Freiburg im Breisgau, Germany; ^2^ Faculty of Biology, University of Freiburg, Freiburg im Breisgau, Germany

**Keywords:** chondrocytes, cell morphology, inflammation, osteoarthritis, projection-based modelling, chondrogenic phenotype, IL-1, cell shape

## Abstract

**Introduction:**

Little is known how inflammatory processes quantitatively affect chondrocyte morphology and how single cell morphometric data could be used as a biological fingerprint of phenotype.

**Methods:**

We investigated whether trainable high-throughput quantitative single cell morphology profiling combined with population-based gene expression analysis can be used to identify biological fingerprints that are discriminatory of control vs. inflammatory phenotypes. The shape of a large number of chondrocytes isolated from bovine healthy and human osteoarthritic (OA) cartilages was quantified under control and inflammatory (IL-1β) conditions using a trainable image analysis technique measuring a panel of cell shape descriptors (area, length, width, circularity, aspect ratio, roundness, solidity). The expression profiles of phenotypically relevant markers were quantified by ddPCR. Statistical analysis, multivariate data exploration, and projection-based modelling were used for identifying specific morphological fingerprints indicative of phenotype.

**Results:**

Cell morphology was sensitive to both cell density and IL-1β. In both cell types, all shape descriptors correlated with expression of extracellular matrix (ECM)- and inflammatory-regulating genes. A hierarchical clustered image map revealed that individual samples sometimes responded differently in control or IL-1β conditions than the overall population. Despite these variances, discriminative projection-based modeling revealed distinct morphological fingerprints that discriminated between control and inflammatory chondrocyte phenotypes: the most essential morphological characteristics attributable to non-treated control cells was a higher cell aspect ratio in healthy bovine chondrocytes and roundness in OA human chondrocytes. In contrast, a higher circularity and width in healthy bovine chondrocytes and length and area in OA human chondrocytes indicated an inflammatory (IL-1β) phenotype. When comparing the two species/health conditions, bovine healthy and human OA chondrocytes exhibited comparable IL-1β-induced morphologies in roundness, a widely recognized marker of chondrocyte phenotype, and aspect ratio.

**Discussion:**

Overall, cell morphology can be used as a biological fingerprint for describing chondrocyte phenotype. Quantitative single cell morphometry in conjunction with advanced methods for multivariate data analysis allows identifying morphological fingerprints that can discriminate between control and inflammatory chondrocyte phenotypes. This approach could be used to assess how culture conditions, inflammatory mediators, and therapeutic modulators regulate cell phenotype and function.

## 1 Introduction

IL-1 plays a central role in the degradation of articular cartilage and in the pathogenesis of osteoarthritis (OA), post-traumatic osteoarthritis (PTOA) and rheumatoid arthritis (RA) (1–5), which are common forms of ‘arthritis’, an umbrella term for a large number of disease processes that lead to whole joint damage, resulting in pain and disability. Chondrocytes as well as other resident joint cells including synovial fibroblasts and pro-inflammatory macrophages produce IL-1 at both an early stage (i.e., prior to clinical disease) as well as during later stages of OA and RA (1, 3). Animal models (6–10) as well as articular cartilage explant and *in vitro* chondrocyte studies have shown that IL-1 exposure has significant effects on the chondrocytes and the joint. IL-1 promotes catabolic activity by suppressing the expression of collagen type II and upregulating the production of aggrecanases and matrix metalloproteinases (MMPs), which degrade the articular cartilage extracellular matrix (ECM), causing the loss of major ECM components including aggrecan and glycosaminoglycans (GAGs). Moreover, IL-1 increases the production of other inflammatory cytokines such as IL-6, IL-8, and leukemia inhibitory factor, a member of the IL-6 family of cytokines, as well as nitric oxide and induces synovial inflammation of the joint (11–23). Several studies have shown that IL-1 production by chondrocytes can result in a self-sustaining positive feedback loop (24–27). This, combined with its ability to induce CD4(+) helper T cell polarization of T cells into IL-17-producing CD4(+) Th17 cells (27), and its synergistic effects with other cytokines (28) locally present in the joint, could sustain a chronic inflammatory response if left unchecked. IL-1 also activates the complement system and the production of chemokines, in turn attracting more inflammatory cells to the joint (29). These studies highlight the relevance of IL-1 in the context of several forms of arthritis.

Within the last few years, high-throughput cell morphometrical methods have emerged and been applied to a wide range of applications (30–32). Quantitative cell shape in particular has become an important aspect of cell phenotype. Indeed, the morphological status of cells can be linked to fundamental cell functions (33) such as cell proliferation (34), cell differentiation (35–39), cell cycle progression (40), cell spreading and cell migration (41), the invasive potential of cancerous cells (42), as well as inflammation (43, 44). Thus, the morphological status of cells can be utilized as a “morphological fingerprint” (30) and quantifying single cell morphology using high-throughput techniques can hence be useful for describing and better understanding the impact of biochemical (45) and biophysical (45–48) stimuli on cells.

While cell morphology has been recognized as a regulator of chondrocyte phenotype (49–54), very few studies actually measured chondrocyte morphology to investigate how inflammatory processes quantitatively affect cell morphology (26, 51, 52), and how this directly relates to phenotypic outcome (51). To the best of our knowledge, only one prior study (51) used cell morphometry to demonstrate a close relationship between quantitative cell shape and, strictly speaking, an abnormal chondrocyte cell shape associated with a fibroblastic and inflammatory phenotype. An abnormal chondrocyte shape in relatively nondegenerate cartilage (Collins grade 0-1 as described in (55)) was depicted as having short (≤5 µm) and more than one cytoplasmic processes per cell. The study (51) demonstrated a close relationship of an abnormal cell shape with a marked increase in cell-associated IL-1β and loss of pericellular type VI collagen. Because the disruption of the functionally relevant pericellular matrix of chondrocytes (56–58) occurs in early OA pathology (59–61), and because IL-1β promotes the sequestration of pericellular type VI collagen (62), the study of (51) is important because it connects chondrocyte morphology with aspects of early articular cartilage degradation and inflammatory processes that revolve, at least in part, around IL-1β.

IL-1β exposure not only induces the well-known *in vitro* dedifferentiation of chondrocytes, it also regulates a number of cytoskeleton-related genes (63–66) and leads to cytoskeletal protein disassembly, affecting multiple downstream signaling effects (67, 68). These mechanisms likely contribute to IL-1β-mediated cell morphology effects such as the increase in cellular volume and cytoplasmic processes (26, 51, 52). Beyond these studies, not much is known about the effects of IL-1β exposure on the quantitative morphology of chondrocytes because the majority of previous studies did not use quantitative single cell morphology descriptors to measure cell morphology, focused only on few shape-related parameters, or quantified a small number of cells. This emphasizes the importance of performing cell morphology-based studies, which could aid in understanding how various conditions such as inflammation modify cell morphology and phenotype and induce or affect the early stages of cartilage degeneration on the cellular level in early OA or other forms of arthritis (53).

In the present study, we performed a feasibility study to investigate how quantitative cell morphology can be used as a biological fingerprint of chondrocytes. We used IL-1β as a representative inflammatory cytokine due to the above introduced reasons, and because IL-1β is a plausible factor in the development of OA, PTOA, and RA (1–5). Using trainable image segmentation, we quantified single cell area, length, width, aspect ratio, roundness, and the number of cytoplasmic processes depicted as a change in cell circularity and solidity. We asked whether such high-throughput quantitative single cell morphology profiling of a large number of cells under control and inflammatory conditions can be combined with cell population-based gene expression analysis (droplet digital PCR; ddPCR) as input into projection-based modelling, and whether this would allow identification of biological fingerprints of control vs. inflamed phenotypes in healthy bovine vs. human OA chondrocytes.

The strength and significance of positive and negative correlations between morphology and gene expression of inflammatory (IL-6 and IL-8) and matrix-regulating (COL1A1, COL2A1, ACAN and SOX9) genes were assessed for control chondrocytes and those under IL-1β exposure. These assessments were compared in bovine healthy and human OA chondrocytes since both are commonly used in *in vitro* studies of OA (1) and this would provide a comprehensive overview of how IL-1β causes morphological and morphologically-related functional effects in chondrocytes in non-diseased and already diseased cells. We then performed a partial least squares-discriminate analysis (PLS-DA) of the measured parameters in which we identified specific cell shape descriptor profiles that associate with non-treated control culture- and IL-1β-induced changes in chondrocyte phenotype. Combining molecular and cell biology with machine learning-based morphology profiling and *in silico* modelling would not only generate a deeper understanding of how chondrocyte morphology relates to phenotype and function, it would also have ramifications for advancing experimental designs that use advanced modeling and bio-image artificial intelligence (AI) methods for cell and tissue explant culture, and, possibly, in *in vivo* applications to utilize immunologically relevant characteristics of single cells and cell populations as quantitative fingerprints.

## 2 Methods

### 2.1 Isolation of chondrocytes from articular cartilage

Human articular cartilage was obtained from the femoral condyles during routine knee replacement procedures in the Department of Orthopedics and Trauma Surgery, University Medical Center Freiburg, Germany. This was conducted with informed written consent according to the guidelines of the Declaration of Helsinki and with approval by the Institutional Ethics Committee of the Albert-Ludwigs-University Freiburg (#418/19). The tissue was transported on the day of the surgical procedure to the laboratory in medium consisting of DMEM (low glucose, GlutaMAX™ Supplement, pyruvate, Thermo Fisher Scientific), 1% 4-(2-hydroxyethyl)-1-piperazineethanesulfonic acid (HEPES, Pan Biotech), 10% FBS superior, 2% penicillin-streptomycin, 2% μg/ml amphotericin B, 0.1% mM nonessential amino acids, 0.4% mM proline and 0.02% L-ascorbic acid phosphate magnesium salt, and briefly stored at 37°C and 5% CO_2_. A comparable procedure was used in our previous studies (58, 69, 70).

Bovine articular cartilage as a source of healthy chondrocytes was harvested from the patella-femoral grooves of 16- to 24-month-old freshly slaughtered cows obtained on the day after slaughter (Emil Färber GmbH & Co. KG, Freiburg, Germany). The knee joint was opened under sterile conditions and the articular cartilage was manually removed from the femoral condyles using a scalpel.

Bovine chondrocytes and, separately, human chondrocytes were isolated overnight for 6 h at 37°C, using 4 ml collagenase XI (1500 U/ml, Sigma Aldrich), dispase II (2.4 U/ml, Sigma Aldrich). The digest was filtered through a 100 µm cell strainer (Thermo Fisher Scientific, Schwerte, Germany). The cell pellet was resuspended in chondrocyte culture medium containing a mix of 1:1 DMEM GlutaMax plus F12 Nut Mix GlutaMax (Thermo Fisher Scientific), with 10% FBS (Biochrom AG, 2% Penicillin-Streptomycin (Life Technologies), 1% Amphotericin B (Biochrom AG) and 0.1% L-Ascorbic acid phosphate magnesium salt (Sigma Aldrich). The cells were incubated at 37°C and 5% CO_2_ until further processing.

### 2.2 Articular cartilage macroscopic grading system

Based on the macroscopic appearance of the articular surface, the articular cartilage explants were graded on a 3-point scale as follows: grade 1 with a macroscopically intact (MIA) surface was characterized by a light pink color with a smooth, even, and shiny surface; grade 2 was characterized by a macroscopically OA (MOA) surface with an uneven, rough, and slightly shiny surface with a slightly darker color than seen in grade 1; grade 3 was characterized by severe MOA identified by an uneven, abrasive, torn or ragged non-shiny surface and a dark pink or reddish color. In total chondrocytes were treated with or without IL-1β, which were isolated from 8 human articular cartilage explants (37.5% MIA, 62.5% MOA) with an average macroscopic damage score of 1.8 ± 0.5 (**Table 1**).

**Table 1:** Macroscopic damage score of human articular cartilage explants used for isolation of OA chondrocytes treated with and without IL-1β.

Donor	Cartilage Grade
1	2
2	1.5
3	2.5
4	1
5	2
6	2
7	1.5
8	2

### 2.3 IL-1β incubation with chondrocytes

All experiments were performed with passage 1 chondrocytes, which were seeded at a cell density of 9375 cells/cm^2^ for human chondrocytes (n = 8 different donors, **Table 1**) and at 3000 or 9375 cells/cm^2^ for bovine chondrocytes (n = 3 different cows performed in duplicate or triplicate). After 24 h, human chondrocytes were treated with 0.1 ng/ml IL-1β (Preprotech), whereas healthy bovine chondrocytes were stimulated with a 0.1 ng/ml or 10 ng/ml of IL-1β. Persistent IL-1β exposure was simulated by adding the cytokine to the media for 6 days, with a media change at day 3. Non-stimulated chondrocytes served as control. Two identical experimental setups were prepared, one for performing marker gene expression analysis and the other for cell staining and subsequent single cell morphological analysis.

### 2.4 Droplet digital PCR for absolute quantification of gene expression

RNA isolation and ddPCR for absolute quantification experiments were performed as previously described in (71). RNA was isolated using the RNeasy Micro Kit (Qiagen) according to the manufacturer’s protocol. The RNA concentration was determined by measuring the optical density at 260 nm. Then cDNA was synthesized from total RNA with oligo(dT) and random hexamer primers by using the Advantage RT-for-PCR Kit (Clontech) according to the manufacturer’s protocol. PCR duplex reactions are performed in 22 µL sample volumes with 11 µL ddPCR Supermix for Probes (no dUTP, Bio-Rad), 1.1 µL of each PrimePCR ddPCR Expression Probe Assay (BioRad) labeled with HEX or FAM, 6.6 µL cDNA with 1.5 ng RNA input and 2.2 µL DNase/RNase-free water. For assessment of the chondrogenic phenotype, the chondrogenic marker genes collagen type II (COL2A1), aggrecan (ACAN) and SOX9 were measured. Chondrogenic de-differentiation and inflammation was assessed by measuring collagen type I (COL1A2), interleukin-6 (IL-6) and interleukin-8 (IL-8) genes. PCR was performed using the QX100 thermal cycler (Bio-Rad) with the following steps. The polymerase activation at 95°C for 10 min, followed by 40 cycles of denaturation at 94°C for 30 s and the annealing at 55°C for 1 min. After cDNA extension, the polymerase was denatured at 98°C for 10 min and the PCR products are kept at 4°C until droplet reading. The fluorescence of the droplets was measured by the QX200 Droplet Reader (Bio-Rad) and analyzed using QuantaSoft Software (Bio-Rad), which quantifies the number of HEX- and FAM-positive and negative droplets and calculates the target concentration for each HEX- and FAM-labeled target gene in copies/µL. Data normalization was achieved by using a standardized amount of RNA for reverse transcription and therefore a standardized amount of cDNA in the reaction volume.

#### 2.4.1 Cell staining and microscopy

To accurately measure single cell morphology, we first stained the chondrocytes after 6 days of incubation with 1 μM calcein (Thermo Fisher Scientific) and 1 μg/ml Hoechst (Thermo Fisher Scientific) to visualize the cell body and nucleus. The cells were incubated in the staining solution for 30 min at 37°C and 5% CO_2_. Then, fresh chondrocyte culture medium was supplied and microscopical images with a 10x magnification were taken with the Axio Observer Z1 microscope (Zeiss Oberkochen, Germany) in a tile format to image entire cell culture wells.

### 2.5 Quantitative panel of cell morphometric shape descriptors

The bioimage analysis tool QuPath (72) was used for converting large whole image samples to a.tif file format and downsizing the images by a factor of three. Then, the images were split into nine single image tiles and out of nine images for each incubation, three representative images were chosen. The selected images were analyzed with an in-house Fiji (73) based single cell shape analysis algorithm tool, which uses the Trainable WEKA Segmentation plugin (74) for segmentation of cells from the background. The WEKA classifier was trained for pixel classification of three classes: nucleus, cytosol, and background. After successful segmentation of the cells from the image background with the WEKA classifier, neighboring cells were separated with a marker-based watershed algorithm. The resulting single chondrocytes were detected in the binary image maps and single cells were assessed by calculating the following shape descriptors: area of the single cells (µm^2^), length (major axis [µm]), width (minor axis [µm]), circularity (4 * π(area/perimeter^2^), aspect ratio (ratio of major to minor axis, used an indicator of cell elongation), roundness (4 * area/(π * major axis length^2^) and solidity (area/convex area(cell)). To clarify, length is different from elongation. Elongation describes an increase in the aspect ratio, which is the ratio between the length and width of a cell. It increases if length continuously increases, while the width either decreases or remains stagnant. Circularity and roundness measurements are relatively insensitive to irregular boundaries, unlike solidity. The solidity measures the density of a cell and is quantified as the ratio of the cell area to the area of a convex hull of the cell. A solidity value of 1 signifies a solid cell, and a value less than 1 signifies a cell with an irregular boundary or a cell containing holes. We had introduced this panel of quantitative shape descriptors originally in the context of mesenchymal stromal cell (MSC) experiments (30, 45, 46, 47, 48, 75) and used the panel here, which allowed testing for correlations between multiple aspects of cell shape with experimental conditions, phenotype, donor and macroscopic grade.

### 2.6 Statistical analysis

The data was analyzed using SigmaPlot v.14.0 (Systat, Chicago) and Microsoft Excel (v. 2013). First, the normality of the data was tested (Kolmogorov-Smirnov-test). For comparing two groups, normally distributed data was subjected to the Student’s t-test and non-normal distributed data was analyzed using the Mann-Whitney-Rank-Sum-test. For comparing more than two statistical groups, an ANOVA on Ranks test was performed. If the ANOVA revealed significant differences between two groups, a *post-hoc* test (Dunn’s Method) was used to compare individual groups. The Dunn’s test allowed comparing groups of unequal sample size. Correlation analyses were performed using the “R” (76) packages “Hmisc” (77) and “corrplot” (78). Spearman Rank Order correlation method was used if one or more variables were categorical. Pearson product moment was used when variables were numerical. For correlation analysis, the control class was coded as 0 and the incubation as 1. To investigate the relationships between chondrocyte morphology and gene expression data in a control and IL-1β environment, a clustered image map (CIM) was generated. This map visualizes scaled and centered data with a color code whose key indicates the standard deviations away from the mean of each feature, whereas dendrograms indicate Euclidian distance-based hierarchical clustering. We also performed multivariate projection-based modelling or partial least squares regression (PLS), on the dataset. PLS-Discriminant Analysis (PLS-DA) is an adaptation of the PLS regression method and was developed to perform classification of categorical data. The resulting PLS-DA loading vectors illustrate the relative importance of each feature. The CIM and PLS-DA analyses of the morphology and gene expression data were performed with the “mixOmics” (79, 80) package in “R”. Statistical differences were considered significant for p<0.05.

### 2.7 Workflow showing how to use cell morphology as a biological fingerprint for describing cell phenotype

**Figure 1:**
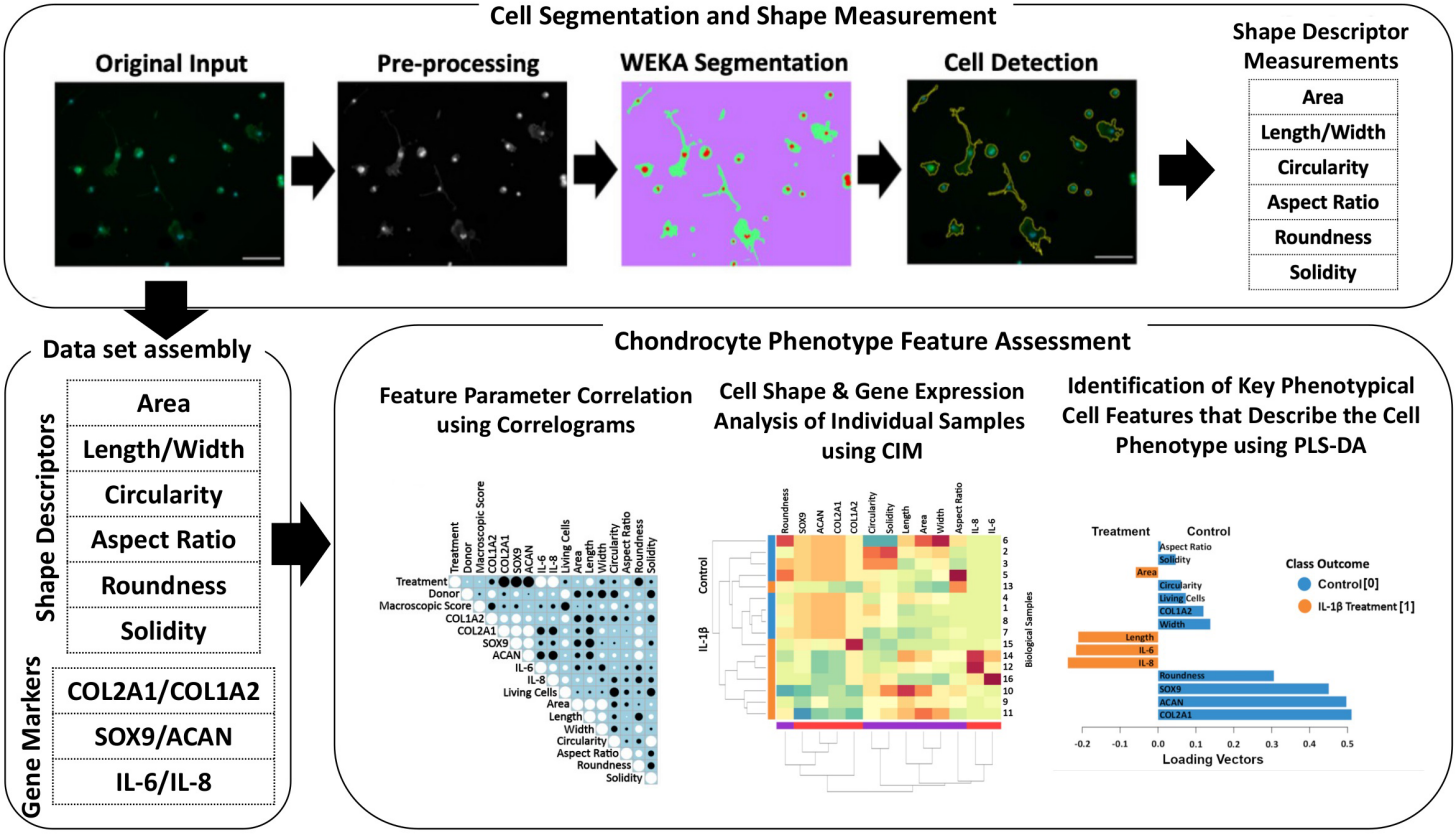
Illustration of the workflow for high-throughput quantitative single cell morphometry in conjunction with multivariate data analysis that allows identification of cell morphological fingerprints. Single cells are first detected using trainable WEKA image segmentation, and then shape descriptors (e.g., area, length, width, circularity, aspect ratio, roundness, and solidity) on a large number of cells are measured. Together with other phenotypic data (e.g., gene expression profiles), the data set is assembled for multivariate cell feature analysis. The relationship between parameters is analyzed using correlograms, CIM, and PLS-DA. Correlograms demonstrate positive and negative relationships across the parameters. CIM can be used to illustrate the expression levels of the features on the biological sample level. Key morphological and other phenotypical cell features, which discriminate between (e.g., control vs. inflammatory) cell phenotypes are calculated using PLS-DA. This multivariate phenotyping approach is applicable to other conditions and cell types and identifies characteristic morphological fingerprints that describe a cell’s phenotype.

An overview of the methods, which allows identification of specific cell morphological fingerprints is provided in **Figure 1**.

## 3 Results

### 3.1 Effects of persistent IL-1β exposure on gene expression and cell morphology in human OA chondrocytes and bovine healthy chondrocytes

**Figure 2:**
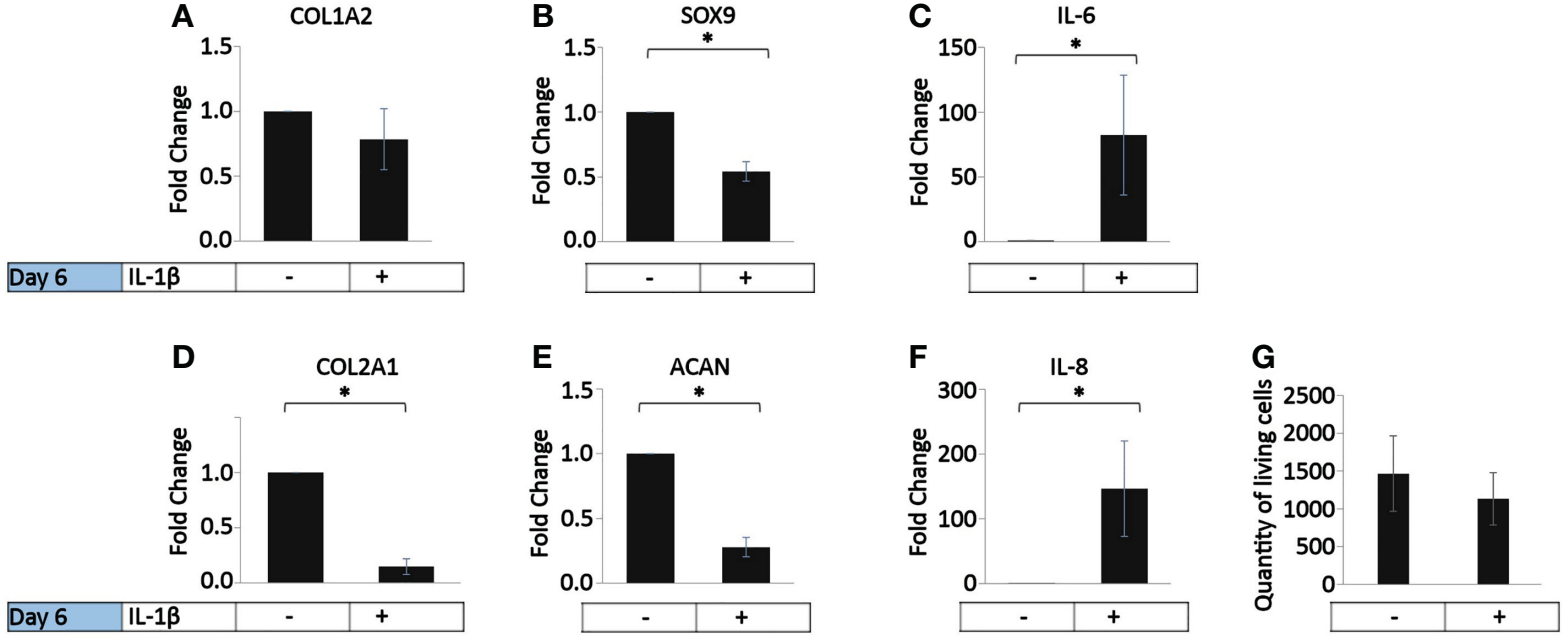
Effect of persistent IL-1β exposure on the marker gene expression of human OA chondrocytes. Human OA chondrocytes were incubated for 6 days with 0.1 ng/ml or without IL-1β (n = 8 per group from 8 different donors). Data is representative of the mean fold change compared to control (without IL-1β) +/- SEM of **(A)** COL1A2, **(B)** COL2A1, **(C)** SOX9, **(D)** ACAN, **(E)** IL-6, and **(F)** IL-8. For fold change vs. control, each control was set to 1. **(G)** quantity of living cells. *p<0.05.

*In vivo*, OA chondrocytes are continuously exposed to IL-1β within the joints (1). Thus, we investigated the influence of the persistent presence of a physiological concentration of IL-1β (0.1 ng/ml) found in the synovial fluid of OA joints (1) on the chondrogenic and inflammatory phenotype of human OA chondrocytes (37.5% MIA, 62.5% MOA, **Table 1**). The cells were incubated with chondrocyte medium supplemented with IL-1β or handled as non-treated control cells for 6 days, with a media change on day 3. Compared to the control, the incubation with IL-1β for 6 days had no effect on COL1A2 expression (**Figure 2A**) or on the number of living cells (**Figure 2G**). In contrast, the incubation with IL-1β resulted in a significant decrease in the expression of COL2A1 (0.1-fold), SOX9 (0.7-fold), and ACAN (0.2-fold) and a significant increase in the expression of IL-6 (82-fold) and IL-8 (147-fold) (**Figures 2B–F**). Thus, in human OA chondrocytes a low dose of IL-1β inhibited the gene expression of healthy ECM production-related genes and promoted the expression of pro-inflammatory genes, indicative of chondrocyte dedifferentiation.

**Figure 3:**
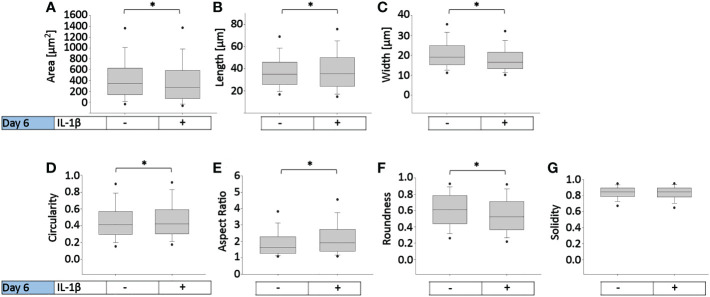
Effect of persistent IL-1β exposure on the cell morphology of human OA chondrocytes. Human OA chondrocytes were incubated for 6 days with 0.1 ng/ml or without IL-1β. Data is representative of **(A)** area, **(B)** length, **(C)** width, **(D)** circularity, **(E)** aspect ratio, **(F)** roundness, and **(G)** solidity values obtained from individually measured chondrocytes with 11733 cells analyzed for the control group and 9058 cells analyzed for the IL-1β incubation group from n = 8 per group isolated from 8 different donors. Box plots: the boxes define the 25th and 75th percentiles, with a central line at the median; error bars define the 10th and 90th percentiles; and dots define the 5th and 95th percentiles.*p<0.05.

To determine how persistent IL-1β influences the cell morphology of human OA chondrocytes, we used a trainable high-throughput method for quantitatively measuring a panel of shape descriptors on a large number of cells. As shown in **Figure 3**, IL-1β incubation significantly decreased the area (**Figure 3A**) and width (**Figure 3C**) of the cells by 13% after 6 days compared to controls. However, IL-1β incubation for 6 days significantly increased the cells’ length and circularity by 2% (**Figures 3B, D**) and the aspect ratio by 17% (**Figure 3E**) compared to controls. Hence, the cells were more cigar-like and elongated after IL-1β incubation. IL-1β also significantly decreased the roundness of OA chondrocytes by 13%, compared to controls but IL-1β did not change the single cell solidity (**Figure 3G**). In summary, IL-1β made human OA chondrocytes smaller, more elongated, less round and more circular indicating fewer or smaller protrusions compared to control cells.

**Figure 4:**
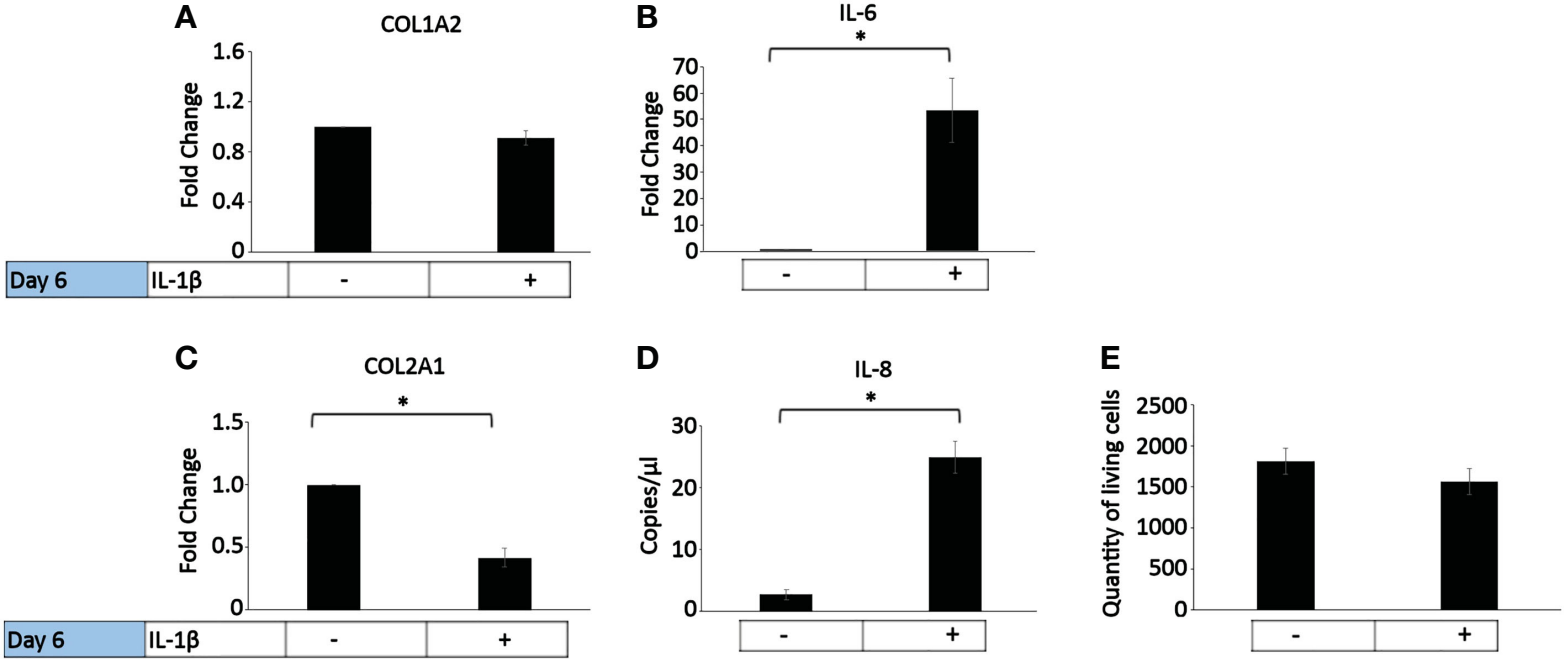
Effect of persistent IL-1β exposure on the marker gene expression of healthy bovine chondrocytes. Bovine healthy chondrocytes were incubated for 6 days with 10 ng/ml or without IL-1β (n = 8 per group isolated from 3 different cows with samples treated in duplicate or triplicate). Data is representative of the mean fold change compared to control (without IL-1β) +/- SEM of **(A)** COL1A2, **(B)** IL-6, and **(C)** COL2A1. For fold change vs. control, each control was set to 1. **(D)** IL-8 is expressed as the mean expression in copies/µl +/- SEM since half of the controls expressed very low levels of IL-8. **(E)** quantity of living cells. *p<0.05.

In healthy individuals, acute inflammation generally occurs for up to 72 h and then generally resolves or persists as chronic inflammation (81). The persistent presence of IL-1β (10 ng/ml) was tested on healthy bovine chondrocytes. The chondrogenic marker gene expression analysis on day 6 (**Figure 4**) showed that IL-1β incubation resulted in a significant decrease in COL2A1 expression (0.5-fold) and a significant increase in IL-6 (50-fold) and IL-8 (22-fold) expression compared to controls. The presence of IL-1β had no effect on the expression of COL1A2 or the number of living cells compared to controls. This suggests that the persistent exposure of healthy chondrocytes to IL-1β negatively impacted their phenotype by inhibiting the gene expression of COL2A1 and by increasing the expression of pro-inflammatory genes. Interestingly, these effects were similar to the effects observed in human OA chondrocytes.

**Figure 5:**
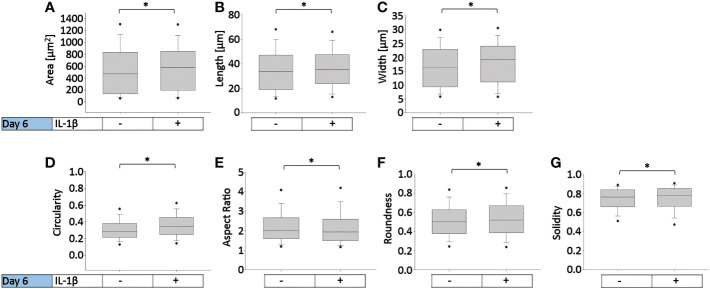
Effect of persistent IL-1β exposure on the cell morphology of healthy bovine chondrocytes. Bovine healthy chondrocytes were incubated for 6 days with 10 ng/ml or without IL-1β. Data is representative of **(A)** area, **(B)** length, **(C)** width, **(D)** circularity, **(E)** aspect ratio, **(F)** roundness, and **(G)** solidity values obtained from individually measured chondrocytes with 1181 cells analyzed for the control group and 2917 cells analyzed for the IL-1β incubation group from n = 8 per group isolated from 3 different cows with samples treated in duplicate or triplicate. Box plots: the boxes define the 25th and 75th percentiles, with a central line at the median; error bars define the 10th and 90th percentiles; and dots define the 5th and 95th percentiles. *p<0.05.

Persistent incubation of healthy chondrocytes with IL-1β significantly increased the cells’ area by 10% (**Figure 5A**), length by 5% (**Figure 5B**), width by 10% (**Figure 5C**), circularity by 15% (**Figure 5D**), roundness by 3% (**Figure 5F**), and the cells’ solidity by 0.6% (**Figure 5G**) compared to controls. Persistent IL-1β decreased the cells’ aspect ratio by 1%, compared to controls (**Figure 5E**). Thus, we observed significant differences in bovine chondrocyte single cell morphology between IL-1β-incubated and non-incubated chondrocytes. This demonstrated that the presence of IL-1β led to profound effects on single cell morphology, as the healthy bovine chondrocytes increased in area, length, width, circularity, roundness and became more solid and less elongated (i.e., their aspect ratio was decreased). The increase in circularity can be understood in this context as indicating smaller or fewer protrusions. These changes were, in part, in contrast to those that observed in human OA chondrocytes (**Figure 3**).

### 3.2 Comparing the cell morphology of healthy bovine chondrocytes in low- and high-density cell culture with different concentrations of IL-1β

**Figure 6:**
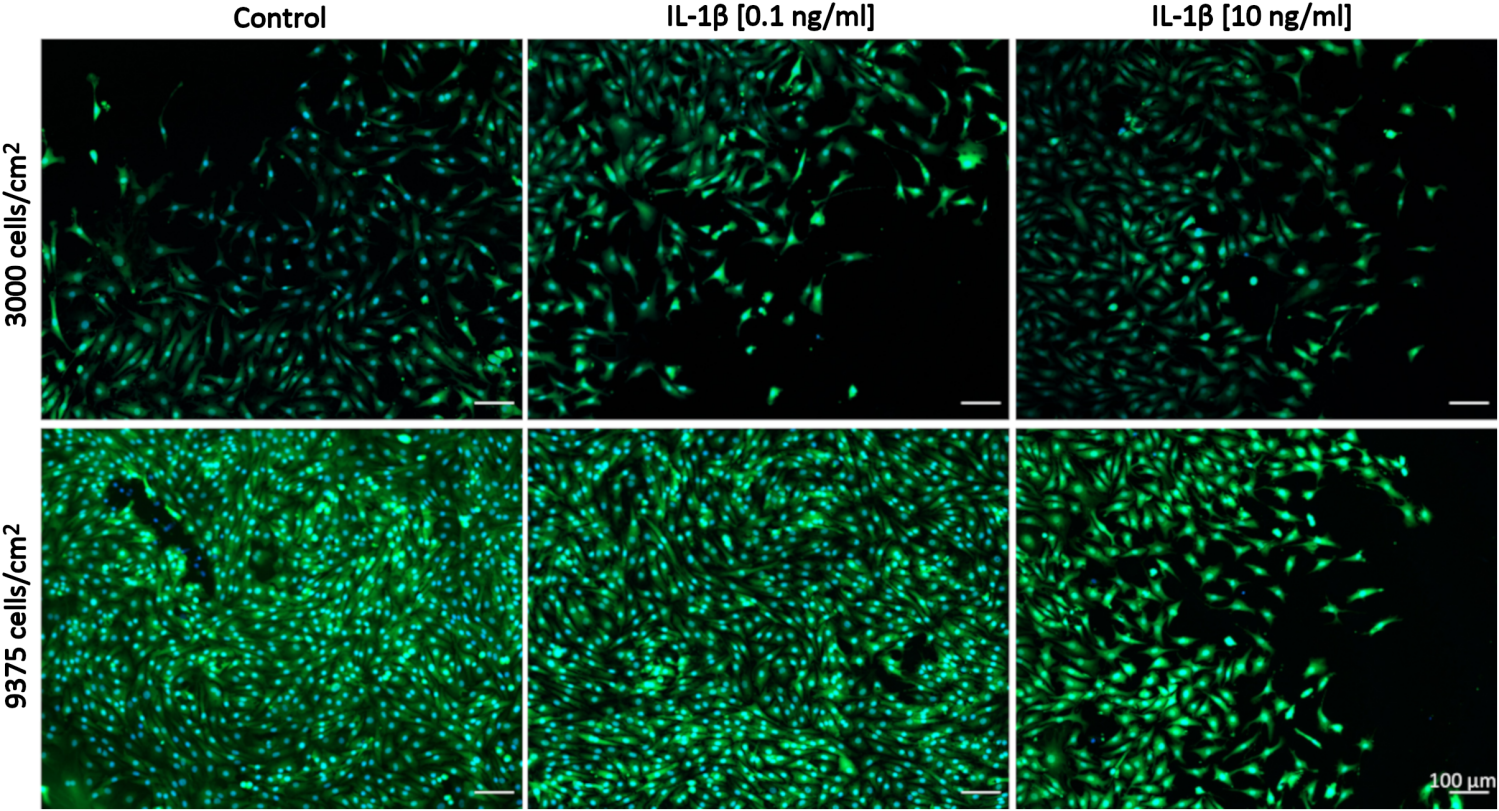
Representative images of healthy bovine single cells. Representative images of healthy bovine chondrocytes in high or low density cell cultures treated with 0 (control), low (0.1 ng/ml), or high (10 ng/ml) doses of IL-1β for 6 days from n = 8 per group isolated from 3 different cows with samples treated in duplicate or triplicate Scale bar, 100 µm.

Comparisons between healthy bovine vs. human OA chondrocytes were initially not intended because we had set out to assess the relative changes between control and IL-1β-exposed cells within each species/model system. We had used different concentrations of IL-1β and had plated cells using different cell densities in OA chondrocytes (**Figures 2**, **3**: 0.1 ng/ml IL-1β and 9375 cells/cm^2^) and healthy bovine chondrocytes (**Figures 4**, **5**: 10 ng/ml IL-1β and 3000 cells/cm^2^). To allow such comparisons between the two, we additionally investigated the cell morphology of bovine healthy chondrocytes in low (3000 cells/cm^2^) vs. high (9375 cells/cm^2^) density conditions treated with 0, 0.1, and 10 ng/ml IL-1β. Representative images of the cells in the various conditions are shown in **Figure 6**. Effectively, this approach allowed subsequently comparing, in the following (3.3) section, the cell morphology of healthy bovine vs. human OA chondrocytes in similar cell density and IL-1β exposure conditions. This additionally answered whether IL-1β concentration and cell density played a role in altering the cell morphology of chondrocytes.

**Figure 7:**
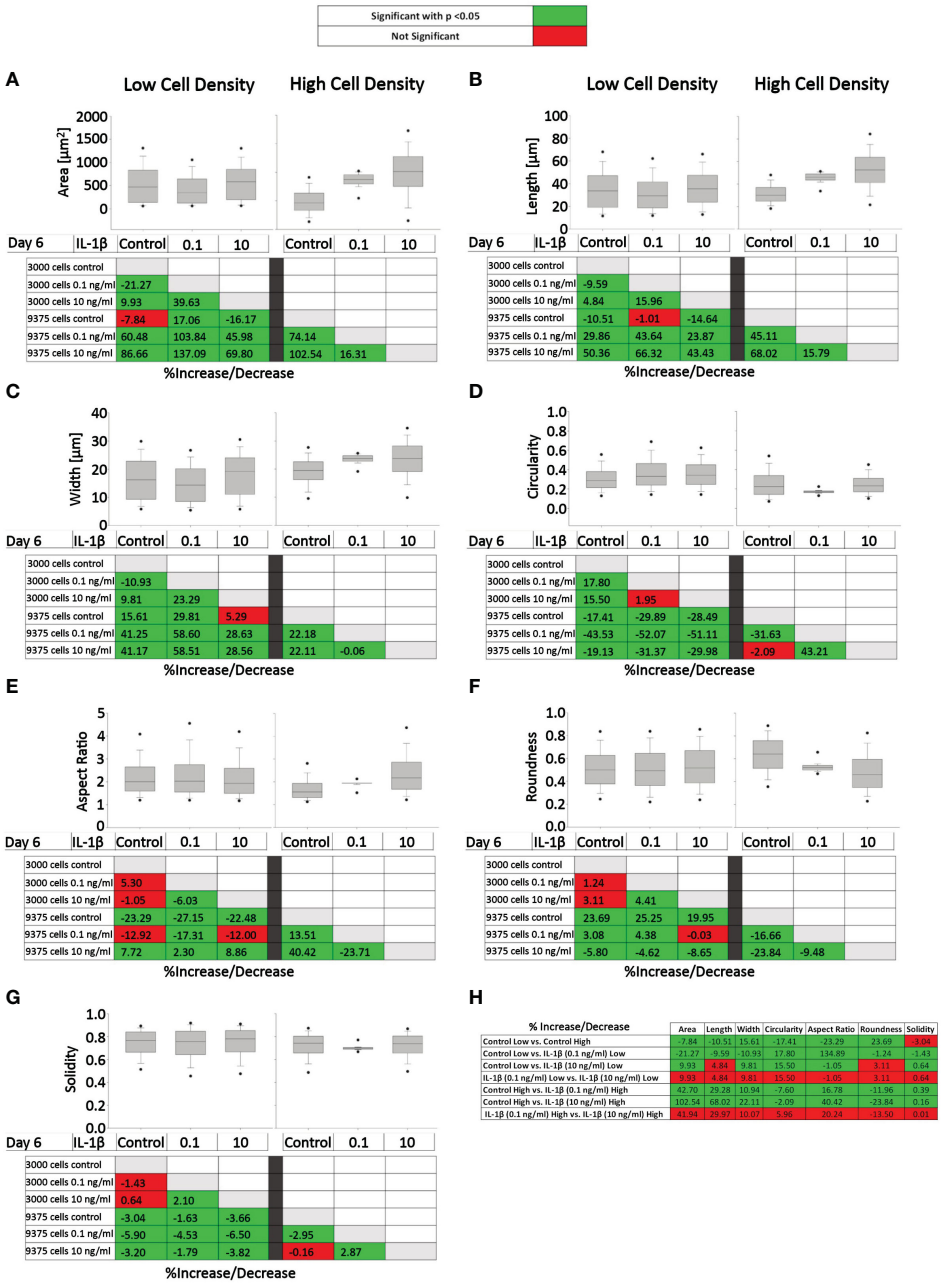
Comparison of healthy bovine single cell morphology under persistent IL-1β conditions in low- and high-density cell cultures. Healthy bovine chondrocytes in high or low density cell cultures were treated with 0 (control), 0.1 ng/ml, or 10 ng/ml of IL-1β for 6 days. Data is representative of **(A)** area, **(B)** length, **(C)** width, **(D)** circularity, **(E)** aspect ratio, **(F)** roundness, and **(G)** solidity with 1181, 2024, and 2917 cells analyzed in the control, 1 ng/ml IL-1β, 10 ng/ml IL-1β-treated cells under low cell density culture conditions and 3604, 2583, and 1923 cells analyzed in the control, 0.1 ng/ml IL-1β, 10 ng/ml IL-1β-treated cells under high cell density culture conditions from n = 8 per group isolated from 3 different cows wit samples treated in duplicate or triplicate. Box plots: the boxes define the 25th and 75th percentiles, with a central line at the median; error bars define the 10th and 90th percentiles; and dots define the 5th and 95th percentiles. In the tables below each of the box plots, the percent changes (increase/decrease) between the specified conditions are provided, with the green color code indicating a significant difference (p<0.05) and the red color code indicating a non-significant difference the two conditions. **(H)** Percent increase/decrease values for each of the shape descriptors comparing the various conditions, with the green color code indicating a significant difference (p<0.05) and a red color code indicating a non-significant difference.

**Figure 7** presents the effects of two IL-1β concentrations in two cell densities for each quantified shape descriptor; the percent increase/decrease for the comparisons are given as color-coded tables beneath the box plots with the green color code indicating changes that reached significant levels between the two indicated conditions. With the exception of modest changes in aspect ratio, roundness and solidity at low cell density (**Figures 7E–G**), this revealed significant differences in cell morphology for almost all shape descriptors across the various conditions investigated vs. controls (**Figures 7A–D**).

To assess whether cell morphology was sensitive to cell density and/or IL-1β concentration, we calculated the percent increase/decrease (**Figure 7H**) values for each of the shape descriptors, which allowed comparing the various conditions; the green color code indicates changes that reached significant levels. We noted that some descriptors, e.g. area and length, showed large % increases without reaching significance (row 7), whereas solidity changed by small but significant amounts (e.g., rows 5 and 6). This can be explained by the range of data for these specific shape descriptors. For example, the boxplots for area (**Figure 7A**) and length (**Figure 7B**) have larger data ranges, while solidity has a relatively small range (**Figure 7G**) and therefore small differences can result in significant differences.

Compared to controls, the greatest significant effects on cell area, length, and width were observed under high IL-1β concentration (10 ng/ml) and high density (row 6), whereas the greatest effects on cell circularity and aspect ratio occurred under low IL-1β concentration (0.1 ng/ml) and low density (row 2). The greatest effects on cell roundness occurred when varying cell density from low to high without IL-1β exposure (row 1) and under high IL-1β concentration and high density conditions compared to controls (row 6). Overall, compared to the control, a high IL-1β concentration in high density conditions had the greatest effect on all shape descriptors, except solidity (row 6). The effects of varying experimental conditions on solidity were small (rows 1-7) but, in some cases, significant.

Surprisingly, varying IL-1β concentration had neither at low (row 4) nor at high density any significant effects (row 7). Hence, no significant changes were observed in any of the shape descriptors when comparing the fold change in morphology from 0.1 to 10 ng/ml IL-1β in both low and high cell density conditions, indicating that IL-1β-induced morphological changes are not related to dosage. Cells became smaller in area and less long and wide in only two conditions: in controls in high cell density compared to low cell density (row 1) and in low cell density with a low IL-1β concentration compared to controls (row 2). Overall, this collectively showed that cell morphology was sensitive to both cell density and IL-1β but varying the concentration of IL-1β had no significant effects.

### 3.3 Comparing the cell morphology of healthy bovine vs. human OA chondrocytes

**Figure 8:**
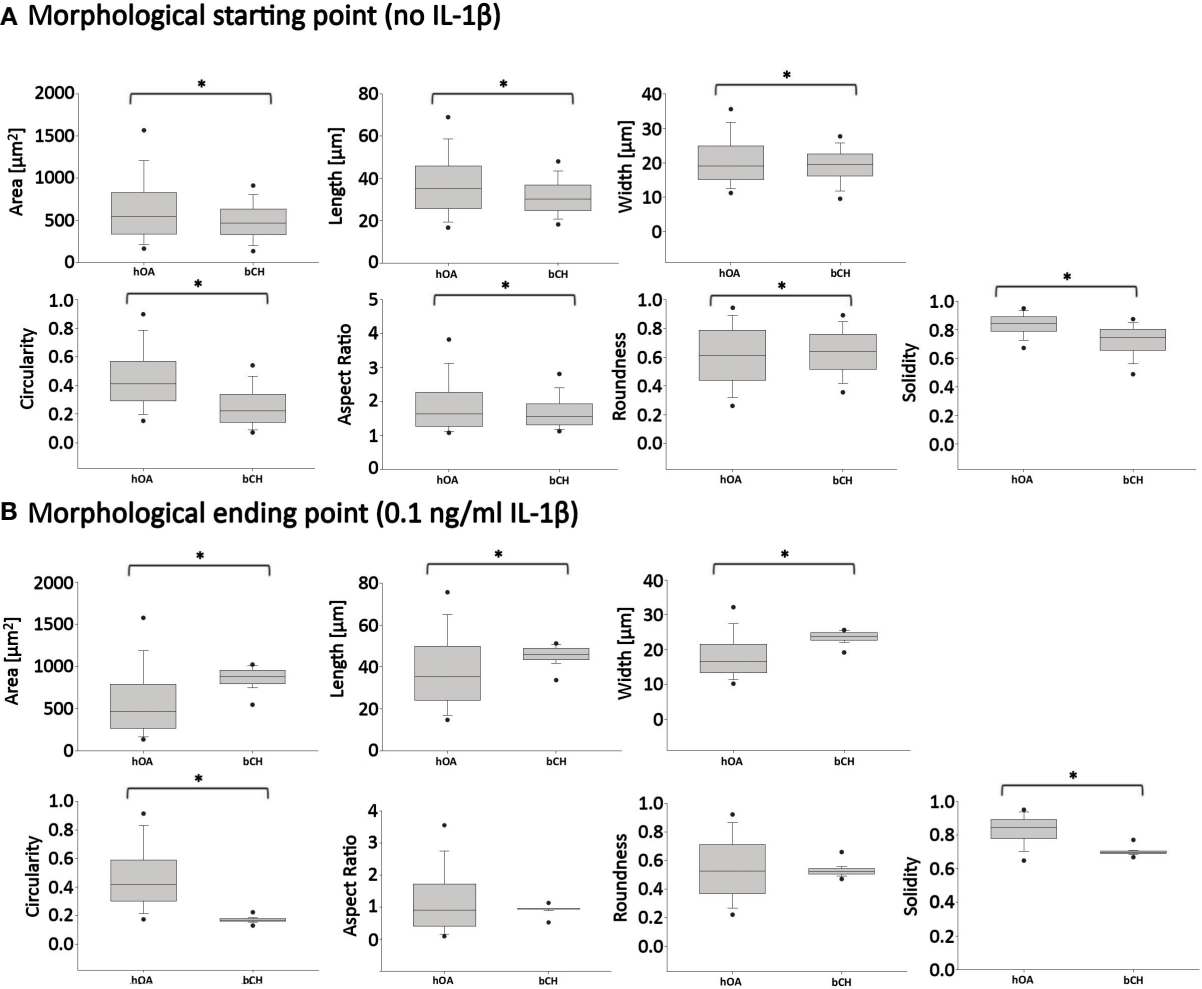
Pairwise comparisons of shape descriptors between human OA vs. healthy bovine chondrocytes under similar high density culture conditions. To allow this side-by-side comparison, data reported in **Figure 2** was used as input human OA (hOA) chondrocyte data and data reported in **Figure 6** was used as input bovine chondrocyte (bCH) data. Day 6 after treatment. **(A)**: hOA chondrocytes were compared to bCHs under control conditions (without IL-1β). **(B)**: hOA chondrocytes were compared to bCHs under low dose IL-1β (0.1 ng/ml) conditions. The horizontal lines represent the median and the boxes show the 25th and 75th percentiles, while the vertical lines represent the 10th and 90th percentiles and the black points illustrate the 5th and 95th percentiles. *p<0.05.

To eliminate the differential density and IL-1β concentration effects when comparing healthy bovine and human OA morphology data, in **Figure 8**, we used the **Figure 7** bovine data, in which cells were cultured under high density conditions and treated with 0 or 0.1 ng/ml IL-1β, for comparisons against human OA chondrocytes (**Figure 3**), as the experimental conditions were matched. Thus, as we already showed, 0.1 ng/ml IL-1β caused healthy bovine chondrocytes to become larger, longer, wider and less round, circular and solid, compared to control cells (**Figure 7**). In contrast, human OA chondrocytes became smaller, more elongated, less round and with fewer or smaller protrusions, compared to control cells (**Figure 3**). While IL-1β treatment clearly modulated many aspects of cell morphology in both OA chondrocytes (**Figure 3**) and healthy chondrocytes (**Figure 5**), IL-1β caused more profound effects in healthy bovine chondrocytes compared to OA chondrocytes (**Figures 8A** vs. **8B**).

What did these differences in the response to IL-1β incubation tell us? To answer this question, we broke the question into two the following parts. First, we compared the ‘starting point’ morphologies of human OA vs. bovine healthy chondrocytes by comparing the untreated control groups (**Figure 8A**). We then compared their ‘finishing point’ morphologies by comparing the IL-1β-treated groups (**Figure 8B**). This revealed that there were significant differences in all shape descriptors between bovine healthy control chondrocytes vs. human OA control chondrocytes (**Figure 8**). This was explained by the fact that the human OA chondrocytes were isolated from OA (already diseased) cartilage with a macroscopic grade of 1.8 ± 0.5 (**Table 1**). Thus, it became apparent that chondrocytes from different health conditions/species exhibited different morphological starting points.

Next, we compared the morphological finishing points (after IL-1β exposure) between the two groups (**Figure 8B**). This revealed that aspect ratio and roundness were not different between the two groups, revealing that the different morphological starting points and responses to IL-1β led to similar aspect ratio and roundness values between the two groups. Since a differentiated chondrocyte is characterized as ‘round’ (67) or ‘spherical’ (68) and, in turn, dedifferentiated chondrocytes lose their round shape, it appears somewhat remarkable that IL-1β incubation led to similar (decreased) roundness values in both groups. When considering morphology as phenotypic marker, these results were in accordance with IL-1β-induced changes in gene expression, namely, that IL-1β inhibited the expression of healthy ECM production-related genes and promoted the expression of pro-inflammatory genes (**Figures 2**, **4**). Other shape descriptors, whose association with phenotype is less clear, were significantly different between healthy bovine chondrocytes vs. human OA chondrocytes. Of those, area showed the largest difference between the two groups, followed by width, length, solidity, and circularity (**Figure 8B**). Thus, despite differences in the morphological starting points and in their relative responses to IL-1β exposure, healthy chondrocytes and human OA chondrocytes exhibited comparable morphological finishing points with regard to roundness and aspect ratio.

### 3.4 Correlation analyses

**Figure 9:**
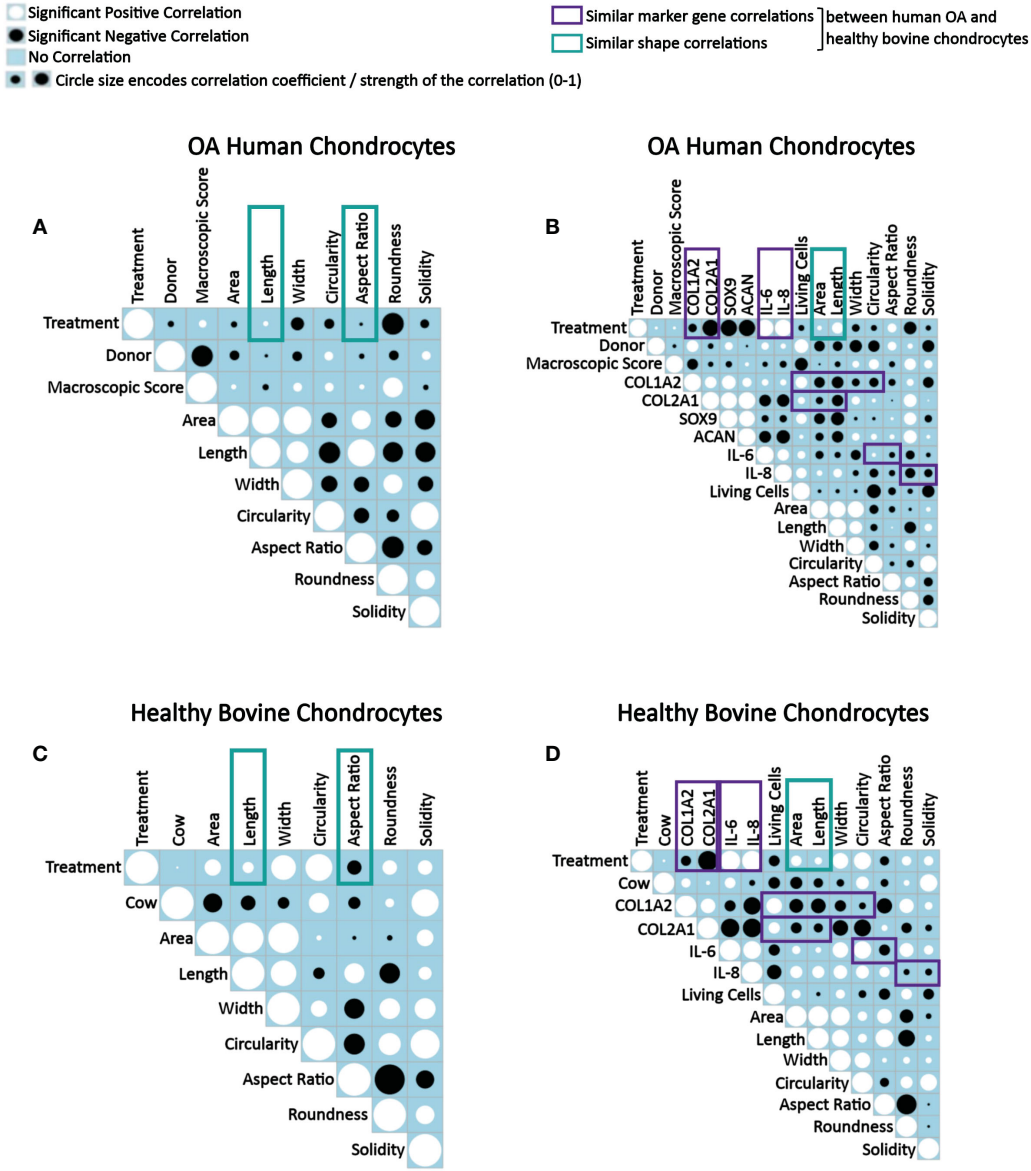
Correlograms depicting correlations between parameters in OA human and healthy bovine chondrocytes across all conditions and highlighting similar correlations found in both cell types. Correlation matrix plots of **(A, B)** human OA chondrocytes treated with or without 0.1 ng/ml IL-1β (n = 8 per group from 8 different donors) and **(C, D)** healthy bovine chondrocytes treated with or without 10 ng/ml IL-1β (n = 8 per group from 3 different cows with samples treated in duplicate or triplicate). Significant positive correlations between the two specified conditions are represented as black circles, while significant negative correlations are represented as white circles (p<0.05). A blue box indicates a lack of correlation. The strength of the relationship is indicated by the size of the circle with a larger circle indicative of a stronger correlation (higher correlation coefficient). **(A, C)** Data is representative of values obtained from individually measured chondrocytes for each of the cell morphology descriptors (n = 20777 OA human chondrocytes from n = 16 control and IL-1β-treated samples; n = 2917 healthy bovine chondrocytes from n = 16 control and IL-1β-treated samples). **(B, D)** Data is representative of the average cell morphology and gene expression values of n= 16 individual experiments for OA human and healthy bovine chondrocytes. The control class was coded as 0 and the IL-1β treatment class was coded as 1. The purple and green boxes indicate similarities in data between OA human and healthy bovine chondrocytes.

As the next step, correlation analyses were performed to understand how IL-1β-induced changes in human and bovine chondrocyte morphology related to the experimental conditions and the macroscopic articular cartilage grade (**Figures 9A, C**), and how those parameters related to changes in the chondrogenic and inflammatory gene expression (**Figures 9B, D**). In **Figures 9A and C**, analyses were performed on data derived from single cells, whereas in **Figures 9B and D**, analyses was performed using the average cell morphology value and the gene expression value measured by ddPCR from each individual experiment. Importantly, correlations were measured across all experimental conditions and not separately for controls or IL-1β conditions in order to detect correlations that occurred while changing from conditions that lacked IL-1β to conditions that had IL-1β present. Interestingly, none of the correlations tested failed to be significant. Below we discuss these significant correlations in detail.

#### 3.4.1 Donor and cell shape descriptor correlations

When assessing correlations between tissue donors and shape descriptors, many correlations in human OA and in healthy bovine chondrocytes were comparable, whereas others were quite different (**Figures 9A–D**). Collectively, these differences illustrated the different morphological starting points and IL-1β responses of the two cell types, in agreement with **Figure 8** as discussed above.

#### 3.4.2 IL-1β incubation and cell shape descriptor correlations

Human OA chondrocytes (**Figure 9B**) and healthy bovine chondrocytes (**Figure 9D**) showed similar correlations between IL-1β treatment and length and aspect ratio (**Figures 9A, C**) and between IL-1β incubation (treatment) and area and length (**Figures 9B, D**). The difference between the panels on the left vs. right side was due to the data level, which explains the subtle differences in the findings. Regardless, the data collectively showed that differences in the morphological response of healthy bovine and OA human chondrocytes to IL-1β reported above in **Figure 8** were found here as well, as indicated by the correlation coefficients.

When collectively reviewing the positive and negative correlations between IL-1β incubation and cell shape descriptors (**Figures 9A, C**), these data showed that IL-1β incubation caused human OA chondrocytes to become less wide, smaller, longer, less round, and less circular. IL-1β treated healthy bovine chondrocytes became larger, longer, wider and more circular, rounder and solid. These correlations were in accordance with the above data (**Figures 3**, **5**, **7**), with the resulting cell shapes consistent with a de-differentiated phenotype.

#### 3.4.3 IL-1β incubation and gene expression correlations

Next, we tested for correlations between IL-1β incubation and resulting gene expression markers (**Figures 9B, D**). In both human OA and bovine healthy chondrocytes, there were a negative correlation between IL-1β treatment and the expression of COL1A2 and between IL-1β treatment and the expression of COL2A1. IL-1β treatment and the expression of IL-6 and IL-8 positively correlated. These similarities were consistent with a de-differentiated phenotype (18, 21, 26, 67). Additionally, in human OA chondrocytes (**Figure 9B**), there was a negative correlation between IL-1β treatment and the gene expression of SOX9 and ACAN. Overall, these correlations (**Figure 9B**) were in line with the changes in gene expression shown in **Figures 2** and **4**. This supported the validity of using this type of approach to assess how persistent IL-1β dynamically changed multifaceted aspects of cell morphology in relation to phenotypic outcome.

#### 3.4.4 Macroscopic cartilage grade, measuring OA-induced damage, and cell shape descriptor/gene expression correlations

Next, the effect of the macroscopic cartilage grade on cell shape and gene expression was analyzed. As shown in **Figure 9B**, there was a negative correlation between the macroscopic cartilage grade and the gene expression of COL1A2, COL2A1, SOX9, IL-6 and IL-8 in human OA chondrocytes, and a positive correlation between the macroscopic cartilage grade and the expression of ACAN. Interestingly, the macroscopic cartilage grade correlated in human OA chondrocytes with all of the cell morphology descriptors measured, which revealed increases in all descriptors except length, which decreased with increasing grade. Thus, aspect ratio and length negatively correlated with macroscopic grade. Area also weakly negatively correlated with macroscopic grade. Macroscopic grade positively correlated with roundness, circularity, width and solidity. This indicates that chondrocytes derived from a higher macroscopic cartilage grade were larger, wider, more circular, rounder but shorter. Correlations regarding the macroscopic cartilage grade were not tested in healthy bovine chondrocytes, as those had all a similarly healthy grade. Moreover, in human OA chondrocytes, the number of living cells correlated negatively with the macroscopic cartilage grade (**Figure 9B**). Together with the shape descriptors circularity and solidity, the negative correlation with the number of living cells suggested that some human OA chondrocytes had died or were dying and, thus, more circular, and solid. This was also seen in healthy bovine chondrocytes (**Figure 9D**). Overall, these data confirmed that OA chondrocytes from more damaged OA cartilage responded differently to IL-1β by exhibiting a larger, wider, more circular, rounder but shorter morphology, compared to chondrocytes from less damaged OA cartilage.

#### 3.4.5 Correlations among gene expression markers

Correlations among gene expression markers showed that in IL-1β-treated human OA chondrocytes, COL1A2 expression positively correlated with COL2A1, SOX9, ACAN, IL-6, and IL-8 expression (**Figure 9B**). The expression of COL2A1 also correlated positively with SOX9 as well as ACAN and COL1A2. Additionally, there was a positive correlation between SOX9 and ACAN. The expression of COL2A1, ACAN and SOX9 correlated negatively with the expression of IL-6 and IL-8, whereas COL1A2 correlated positively with the IL-6 and IL-8 expression. The IL-6 expression positively correlated with IL-8 expression. These findings, including that COL2A1 negatively correlated with IL-6 and IL-8, were comparable to healthy bovine chondrocytes (**Figure 9D**) except that IL-6 and IL-8 expression positively correlated with COL1A2 expression in human OA chondrocytes and negatively in healthy bovine chondrocytes. In both cell types, COL1A2 and COL2A1 expression positively correlated with one another. The findings generally agreed with what is known about healthy and inflamed chondrocytes (1).

#### 3.4.6 Gene expression and cell shape descriptor correlations

We then tested for correlations between the gene expression markers and shape descriptors. In both OA chondrocytes (**Figure 9B**) and in healthy bovine chondrocytes (**Figure 9D**), the expression of COL1A2 positively correlated with cell roundness and negatively with cell area, length, width, circularity, and aspect ratio. For solidity, COL1A2 negatively correlated in OA chondrocytes (**Figure 9B**) but positively correlated in healthy bovine chondrocytes (**Figure 9D**). While COL2A1 positively correlated in human OA chondrocytes with width, circularity, roundness, and solidity and negatively with area, length, and aspect ratio (**Figure 9B**), COL2A1 positively correlated in healthy bovine chondrocytes with the aspect ratio of the cells and negatively with area, length, width circularity, roundness, and solidity (**Figure 9D**). Changes in area and length correlated similarly and negatively with the COL2A1 expression. Thus, COL1A2 expression correlated similarly with six out of seven shape descriptors in both human OA and healthy bovine chondrocytes, whereas COL2A1 expression correlated similarly with only two out of seven shape descriptors in both human OA and healthy bovine chondrocytes. This revealed gene-specific differences in the correlation patterns with cell shape descriptors, which was even more obvious for IL-6 and Il-8. Collectively, these extensive correlation analyses supported the presence of differential morphological starting points and responses of healthy vs. diseased chondrocytes. Moreover, these analyses revealed complex, correlated changes, in which an IL-1β inflammatory environment decreased the gene expression data of COL2A1 and COL1A2, increased the levels of IL-6, and IL-8 expression in both OA human and healthy bovine models, and co-occurring morphological changes that were indicative of a dedifferentiated phenotype, whose complexity was further analyzed as described below.

### 3.5 Identifying key cell shape features that discriminate between control vs. IL-1β conditions

**Figure 10:**
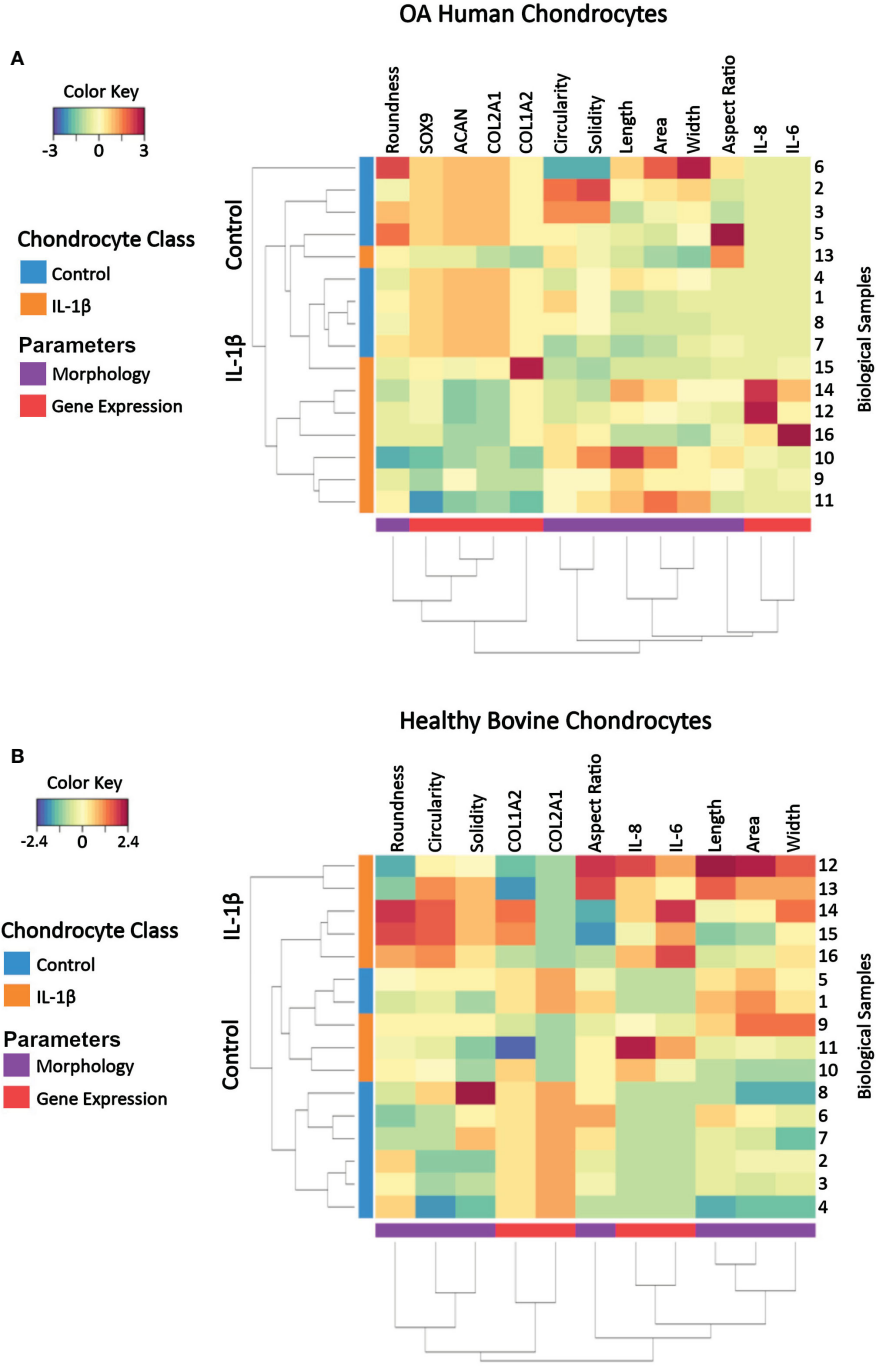
Clustered image map of cell morphology and gene expression values from individual samples of **(A)** OA human and **(B)** healthy bovine chondrocytes that were untreated or treated with IL-1β. Each horizontal row represents 16 biological samples treated with or without IL-1β as indicated in the figure (n=8 per group isolated from n = 8 different OA donors and n = 3 healthy bovine cows with samples treated in duplicate or triplicate). Rows are clustered using cell morphology and gene expression and color-coded according to experimental conditions: control cells vs. IL-1β incubation. A CIM visualizes scaled and centered data, with a color code that indicates the standard deviations away from the mean of each feature, whereas the dendrograms indicate clustering. The level of the parameters of a given category and their intensity of the red color denotes the number of standard deviations above the overall mean across all samples and intensity of the blue color denotes the number of standard deviations below the overall mean representing increases and decreases in the markers from the overall mean category. Based on the multi-feature signatures, the CIM revealed a discrimination of the control vs. IL-1β and revealed signature patterns.

A clustered image (CIM) map visualizes patterns of similarity vs. dissimilarity in multivariate data, which can be used to explore data at the sample level relative to experimental conditions. Dendrograms indicate Euclidian distance-based hierarchical data clustering and the height of the dendrogram represents the distance between clusters (82). The here used CIM was constructed by using scaled and centered data on healthy bovine vs. human OA chondrocytes whose color key indicates the standard deviations away from the mean. The CIM presents the experimentally determined multivariate dataset of control and IL-1β-treated human OA chondrocytes (**Figure 10A**) and healthy bovine chondrocytes (**Figure 10B**).

In both human OA chondrocytes and healthy bovine chondrocytes, the main clusters almost perfectly distinguished the experimental conditions (controls vs. IL-1β incubation). In human OA chondrocytes only one IL-1β treated sample (sample 13) clustered as a non-treated control sample. For healthy bovine chondrocytes only two control samples (samples 1 and 5) clustered with the IL-1β treated group. Although these two samples were similar to the control cluster in their gene expression of COL2A1, COL1A2, IL-8, and IL-6, the morphology descriptors length, area, and width were more comparable with IL-1β treated group. In human OA chondrocytes not all samples responded equally to IL-1β incubation: the color pattern differed in samples 16, 12, and 14 compared to samples 11, 9, and 10 in SOX9, COL1A2, IL-6 and IL-8 expression but also in cell width. This showed that, in some cases, individual samples could be different from the overall population. This highlighted the importance of measuring multiple parameters to distinguish between phenotypes. In turn, this indicated that, using this panel of measured parameters, cell morphology and gene expression profiles are sufficient to characterize control and inflammatory phenotypes.

The CIM also illustrated the changes from the overall mean and from this, general relationships between morphology and gene expression can be visualized in individual samples. Under control conditions, with the exception of up to a few individual samples, bovine healthy chondrocyte samples had a lower cell circularity and higher expression of COL2A1 compared to the overall mean (**Figure 10B**), whereas OA chondrocytes were rounder and the expression of healthy marker genes COL2A1, ACAN, and SOX9 *w*as higher than the overall mean (**Figure 9A**). Under IL-1β conditions, bovine healthy chondrocyte samples were higher in circularity and width with a higher expression of the inflammatory genes IL-6 and IL-8 and lower expression of COL2A1 compared to the overall mean. OA chondrocytes were longer with a higher expression of the inflammatory genes IL-6 and IL-8 and lower expression of COL2A1, ACAN, and SOX9 than the mean (**Figure 10A**).

Thus, the CIM supported the above presented correlation analyses (**Figure 9**) but also revealed differences across the samples within a given experimental group that were not visible in the correlation analysis results. This meant that phenotypic features such as morphology and gene expression broadly followed the experimental conditions but also displayed certain variations, which correlation analyses had traced back in part to parameters such as the individual donor/cow, among other parameters. In turn, this meant that a phenotypical fingerprint derived from this multivariate dataset begun to emerge but needed another method for exact determination.

To determine which of the measured shape features provided the most value in discriminating between control vs. IL-1β conditions in the two different cell types (healthy vs. OA) we used projection-based modeling. PLS-DA is a multivariate dimensionality-reduction tool (83–85) that weighs each feature and the above CIM-visualized variability of the response for discriminating groups (e.g., IL-1β incubation vs. control). One of the outputs of the model was the relative importance of each feature for discrimination, which is derived from the model loadings. Therefore, PLS-DA was used to identify key variables that discriminate between non-incubation controls and IL-1β-stimulated human OA and healthy bovine chondrocytes. The length of the bar corresponded to the loading weight and, thus, importance of each assessed feature.

**Figure 11:**
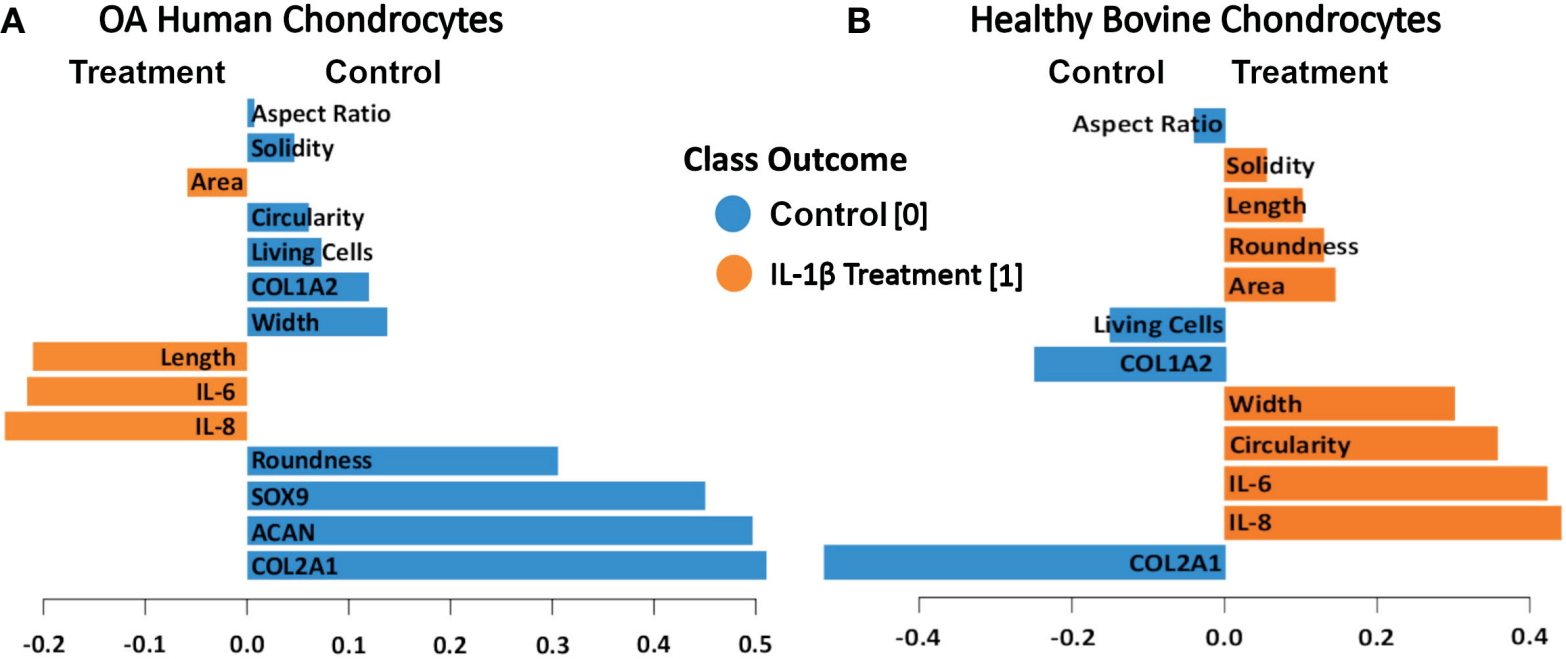
PLS-DA of cell morphology descriptors and chondrogenic and inflammatory gene expression markers in **(A)** OA human and **(B)** healthy bovine chondrocytes. We used scaled data from **Figure 8** for the PLS-DA. The length of the loading vector bars of the PLS-DA plots, represent the importance of a feature to discriminate between control and IL-1β incubation phenotypes. All data reported in **Figure 8** was used as input data using the averages of the data. The control classes were coded as 0 and the IL-1β treated classes were coded as 1.

The PLS-DA analysis of human OA chondrocytes (**Figure 11A**) revealed that IL-1β treated chondrocytes could be discriminated from control cells by their gene expression levels of IL-8 and IL-6. The non-stimulated control-treated cells were discriminated based on their expression of COL2A1, ACAN, and SOX9. More crucially, the length and area of the cells were critical in discriminating IL-1β-treated OA chondrocytes, whereas chondrocyte roundness was the most essential shape descriptor in identifying control OA chondrocytes. For healthy bovine chondrocytes (**Figure 11B**) the PLS-DA showed that IL-1β-incubated chondrocytes could also be discriminated from control cells by their gene expression levels of IL-8 and IL-6, whereas the control cells were discriminated based on their expression of COL2A1 and COL1A2 (**Figure 11A**). Importantly, the chondrocyte aspect ratio was most indicative for control cells and circularity and width for the inflammatory phenotype. This was principally in line with the results of the hierarchically clustered CIM (**Figure 11**), reinforcing that we have identified specific morphological fingerprints that are capable of discriminating between chondrocyte phenotype in control and inflammatory conditions.

## 4 Discussion

The present study demonstrated that cell morphology can be used a biological fingerprint for describing chondrocyte phenotype. Using IL-1β as a representative inflammatory cytokine, we showed that it is possible to use quantitative single cell morphometry in conjunction with multivariate projection-based modelling for identifying specific morphological fingerprints that discriminate between experimental conditions. Using scaled data from the correlation data (**Figure 9**), we identified the morphological characteristics attributable to control and IL-1β-induced inflammatory phenotypes (**Figure 11**). This approach is technically applicable to other cell types and conditions using the approach described in **Figure 1**.

The here-derived insights substantially extend the currently available knowledge of chondrocyte morphology since only a few studies *i)* recognized cell morphology as a regulator of chondrocyte phenotype (49–54) and *ii)* connected chondrocyte morphology to pathology, e.g. passage-induced dedifferentiation (86–90), early articular cartilage degeneration (51, 53), or IL-1β exposure (26, 51, 52). These studies revealed that early passaged *in vitro* cultured chondrocytes show a characteristic single cell shape with a round and spheroid morphology, a small diameter (86), a small spreading area, and a low aspect ratio (87). Chondrocytes in passage 2 and higher assume an amoeboid and fibroblast-like shape (86–89) with a large diameter (86), spreading area and elongation factor (87), and reduced number of primary cilia (90). *In vitro* and *ex vivo*, IL1-β increases the cellular volume (26, 52) and, *ex vivo*, the number of cytoplasmic processes per cell (51). Our data detailed multi-dimensional aspects of chondrocyte morphology, the relationship between cell morphology and gene expression as well as OA macroscopic grade, the effect of cell density, IL1-β concentration, species and disease state on cell morphology, proving with the here established method that cell morphology can be used to describe chondrocyte phenotype.

IL-1β plays a major role in OA, PTOA and RA (1–5) but only a few studies quantitatively demonstrated a relationship between IL-1β and cell morphology (26, 51, 52). We investigated how control conditions and persistent IL-1β affected the phenotype of passage 1 OA human and healthy bovine chondrocytes. We identified novel relationships between single chondrocyte morphology parameters and marker gene expression profiles that provide causal evidence how to interpret previously shown associations between cell morphology, IL-1β, and early degeneration (51). IL-1β incubation caused considerable alterations in chondrocyte shape, which was linked to changes in the expression of ECM- and inflammatory-regulating genes. Next, we asked if cell morphology could be used for discriminating chondrocyte phenotype. Applying CIM and PLS-DA, we demonstrated that the here chosen experimental and analytical approach (**Figure 1**) allowed identifying, for the first time, those shape features that are most important for discriminating control vs. pro-inflammatory chondrocyte phenotypes. The specific morphological fingerprints revealed by PLS-DA feature importance (**Figure 11**) were roundness and width as cell shape discriminators of a healthier phenotype of human OA chondrocytes and cell length and area as discriminators of a more inflamed phenotype of human OA chondrocytes, here induced with IL-1β. In healthy bovine chondrocytes, circularity and width discriminated an inflamed phenotype, whereas cell aspect ratio discriminated control cells.

In this feasibility study, we used IL-1β as a representative cytokine to demonstrate that trainable, automated high-throughput image analysis techniques of single-cell morphology can be combined with population-based gene expression analyses by ddPCR. Despite being one of the major cytokines, IL-1β alone cannot imitate the high complexity of joint inflammation and investigations on other inflammatory triggers that play an important role in joint inflammation such as other types of inflammatory cytokines (1) or monosodium urate or calcium pyrophosphate crystals (91) are needed. Moreover, inflammation in the joint can be acute or persist for weeks to months or longer (1). Another consideration is that 3D and *in vivo* environments are far more complex than 2D environments, which may imply that the absolute measurement values obtained in this study in 2D might be different in a 3D environment. Future experiments would need to address these points to determine the morphological fingerprint induced by those conditions.

Another consideration is that there may be potential cartilage zone-dependent or other (e.g., biomechanical) effects on cell shape throughout the tissue depth and that there may be differences in chondrocyte shape across zones in healthy vs degenerate cartilages. Here, we used chondrocytes isolated from bulk articular cartilage samples. In healthy cartilage, superficial zone (SZ) chondrocytes are elliptical in form but round in deeper zones (92). With increasing cartilage degeneration, chondrocyte volume and the number and length of cell processes increase significantly within the SZ but not in the deep zones (93, 94). The degradation of collagen in the SZ also modulates chondrocyte volume and morphology in relation to mechanobiological properties of cartilage (95). Since our study contained a mixture of cells from all cartilage zones, the cell morphology results depicted the averages of the entire cell populations that were isolated from the articular cartilages. Since human OA chondrocytes were isolated from OA cartilage (macroscopic grade of 1.8 +/- 0.5) and may have, in part, lost some of its SZ (96), whereas healthy cartilage contained an intact SZ, this may have potentially increased the cell length values, due to the elliptical morphology of SZ chondrocytes in the healthy chondrocyte data. We showed that the average cell length (major axis) of bulk healthy chondrocytes under control conditions was 35 µm. Considering that 32% of intact healthy cartilage is made of SZ chondrocytes (92), we calculated that the inclusion of SZ chondrocytes may have shifted the average cell length of healthy bovine chondrocytes reported here up but only by a maximum of 7 µm (but not in the human data because the SZ may have been lost to some degree). Interestingly, Murray et al. (51) demonstrated by fluorescence immunohistochemistry of chondrocytes within *ex vivo* explants that abnormal morphology was associated with increased cell-associated IL-1β levels. Chondrocytes from the SZ have approximately twice the number of IL-1 receptors in comparison to cells from the deeper layers of the same joint (97). Therefore, assuming that IL-1β alters chondrocyte morphology, IL-1β may not equally induce changes in all cells from different zones. In our present study, we demonstrated that IL-1β altered chondrocyte morphology in a cell density-dependent way, hinting that abnormal morphology associated with IL-1β levels in degenerate cartilage may have been caused by IL-1β; although the alternative, that morphology affected IL-1β levels, would remain possible. Another point to consider is that articular cartilage chondrocytes isolated from the knee are more susceptible to IL-1β-mediated cartilage degradation effects than chondrocytes isolated from the ankle (98, 99). While this was not an issue in our study since both human and bovine chondrocytes were isolated from the knee joint, the presence of the SZ in healthy bovine cartilage and potentially partial absence in human OA cartilage must be considered when assessing the data from bulk cartilage samples. Therefore, our future studies will include zonal and causal aspects to determine how these topics are related.

Previous research demonstrated that 0.1 ng/ml is close to the physiological relevant concentration of IL-1β during joint inflammation in the human knee joint (1). Hence, this dosage was used in a 6 day persistent inflammatory OA human chondrocyte model. In response to persistent IL-1β, the chondrogenic gene expression of COL2A1, SOX9 and ACAN decreased and the expression of the inflammatory genes IL-6 and IL-8 increased and significantly changed their chondrocyte morphology. The cells became significantly smaller, decreased in cell length, width, roundness, and solidity, but increased their circularity and aspect ratio. The increase in aspect ratio as well as the decrease of cell roundness and solidity were indicative of chondrocyte de-differentiation, which was confirmed by the decrease in COL2A1, SOX9 and ACAN expression. Other studies have also shown that IL-1β increases the expression of IL-6 and IL-8 and decreases the expression of healthy chondrocyte phenotype markers ACAN and COL2A1 (18, 21, 26, 67, 100), indicating that the present study’s gene expression results were in line with the literature. Interestingly, there was no change in COL1A2 expression, which could be due to the fact that OA is irreversible, and the chondrocytes were isolated from already diseased tissue. The correlation analyses showed a relationship between the shape of chondrocytes and their gene expression, as the expression of COL2A1, SOX9 and ACAN positively correlated with cell roundness and the expression of SOX9, but negatively with cell length in OA chondrocytes. In agreement with other studies (51, 93), the expression of IL-6 and IL-8 was connected to the chondrocyte size and length indicative of a fibroblastic de-differentiated cell phenotype. These findings showed that persistent IL-1β further promoted a de-differentiated phenotype on both the cell morphology and gene level. Interestingly, this occurred despite the fact that human chondrocytes were isolated from articular cartilage having a macroscopic grade of 1.8 +/-0.5, which is considered somewhere between macroscopically intact articular cartilage and macroscopically OA lesion articular cartilage. In the context of OA on the tissue level, such processes might enhance progression towards full OA and/or promote the “healthier” parts of the cartilage tissue to become diseased.

In this study, a higher concentration of IL-1β (10 ng/ml) was used in healthy bovine chondrocytes. Persistent IL-1β significantly decreased the gene expression of COL2A1 and elevated the expression of the inflammatory genes IL-6 and IL-8. While we did not measure SOX9 and ACAN in the bovine study, others have shown that persistent IL-1β can significantly decrease their gene expression (101–103). Similar to other studies, IL-1β significantly decreased the expression of COL2A1, and increased IL-6 and IL-8 (23, 104). Moreover, our data is in line with changes in cell volume that others have already quantitatively shown to be altered in chondrocytes by IL-1β. We observed a change in chondrocyte cell volume (as measured by area), a known marker of de-differentiation (53), which increased in healthy chondrocytes, in line with IL-1β-mediated increases in cell volume as shown in healthy chondrocytes *ex vivo* and *in vitro* (26, 52). Interestingly, in healthy bovine chondrocytes, differential IL-1β-induced morphological effects were observed in comparison to human OA chondrocytes. In healthy bovine chondrocytes, IL-1β increased the area, length, width, roundness, and solidity, whereas the aspect ratio of the cells was decreased. The correlation analyses between gene expression markers and cell morphology descriptors showed that cell area, length, roundness, solidity, but especially circularity and cell width positively correlated with the expression of IL-6 and IL-8. Interestingly, aspect ratio positively correlated with the expression of IL-8, but negatively with IL-6. Hence, the persistent presence of IL-1β led to changes in chondrocyte morphology and gene expression of healthy chondrocytes. This suggests that IL-1β could promote disease progression in healthy chondrocytes under early inflammatory stimuli by modifying cell morphology.

Interestingly, when we analyzed cell shape descriptors across conditions or species, we noted apparent differences in the morphological response of the cells to IL-1β. Thus, IL-1β induced human OA chondrocytes to become smaller, more elongated, less round and more circular (indicating fewer or smaller protrusions), whereas IL-1β led in healthy bovine chondrocytes to increased area, length, width, circularity, roundness and solidity, and less elongation. We resolved these differences in the response, which were initially difficult to understand, conceptually. We introduced the idea that chondrocytes from different health conditions/species exhibited different morphological starting points and subsequently different responses to IL-1β. Those led to morphological finishing points, in which aspects of cell shape known to be phenotypically relevant were, surprisingly, statistically equal: cell roundness and its inverse term, aspect ratio. Hence, IL-1β incubation produced equally round and elongated bovine and human cells, despite differences in species and the state of tissue health. Therefore, while we saw differential effects of IL-1β on already diseased human OA chondrocytes (that were enhanced to a fully de-differentiated phenotype by IL-1β) vs. healthy bovine chondrocytes (that were moving towards a de-differentiated phenotype), it clearly shows that IL-1β plays a major role in modifying the cell morphology and gene expression of both healthy and diseased cell types.

Due to the limited access to healthy human cartilage, bovine cartilage was used in the present study. While it is known that species-related cartilage differences exist (e.g., bovine cartilage has a decreased thickness and a higher cellularity vs. human cartilage (105)), the development of naturally occurring OA in bovine joints very closely mimics the onset and progression of OA in humans (105). We do not believe that the differences are due to a difference in species per se but instead it is likely due to the “inflammatory heritage” of the chondrocytes. It is an already known fact that OA cartilage comes from an inflamed environment (1) and the quality of cartilage can vary for from donor to donor. The lack of access to healthy human or bovine OA cartilage tissue of the exact same grade (1.8 +/- 0.5) limits us to draw a general applicable conclusion. To rule out dosage differences we plated healthy bovine chondrocytes in a low and high cell density and treated the cells with either 0.1 ng/ml or 10 ng/ml IL-1β. The results showed that changes in cell density and IL-1β incubation, but not IL-1β concentration, clearly affected single-cell chondrocyte morphology. However, regardless of the starting point (i.e., the lack of disease or disease severity), we demonstrated that chondrocytes changed their cell morphology in response to IL-1β. This is advantageous, because it shows that single cell morphology descriptors are sensitive to changes and can be used to discriminate between different phenotype classes and culture conditions.

It is important to note that the significant but sometimes small percent changes in morphology observed here, induced by IL-1β, were associated with large phenotypic effects, e.g. the expression of COL2A1 and IL-6 (**Figures 2**, **4**), which highlights that small changes in morphology cannot be dismissed because of their small scale. In this context, we have reported comparable findings in MSCs induced by biomechanical forces (45–47), biophysical stimuli including biomaterials and nanoscale surface stiffness (46), implant surface topography (48) and growth factors (45), highlighting that small changes in morphology are related to large phenotypic effects. In term of the underlying mechanisms of cytokine-mediated cell morphology and associated gene effects, we refer the interested reader to our recent review (67) that discusses how inflammatory cytokines such as IL-1β and other factors regulate articular chondrocyte phenotype through the cytoskeleton and the signaling processes that originate from or converge on the cytoskeleton. Specifically, pro-inflammatory cytokines including IL-1β as well as IL-1α, TNF-α, IL-6 and IL-8 (21, 23, 106), which are all involved in joint inflammation (1), promote signaling of RhoA/ROCK/Rac1/mDia1/mDia2/Cdc42 and stress fiber formation (107–109). This can in turn control the morphology and phenotype of chondrocytes. To illustrate, inhibitors of these signaling pathways such as ROCK inhibitors modulate the cell morphology of chondrocytes into a rounder morphology that leads to cortical actin organization (110) and increased SOX9, ACAN, and COL2A1 gene expression (110–113). This highlights that changes in chondrocyte morphology due to alterations in cytoskeletal elements and their second messenger pathways can consequently affect chondrocyte cell function and perhaps even joint health through, e.g., production of a mechanically weak matrix, which could further promote disease progression (53, 67), but that more in-depth studies are needed.

This study performed a thorough analysis of how IL-1β alters chondrocyte gene expression and morphology of human OA and healthy bovine chondrocytes. We showed that the significant changes in chondrocyte morphology caused by IL-1β incubation can be correlated to changes in gene expression of healthy matrix-regulating chondrogenic and inflammatory genes. While IL-1β led to less of a de-differentiated cell shape in healthy chondrocytes vs. OA chondrocytes, IL-1β caused early morphological effects as well as phenotypical effects in non-diseased, previously healthy chondrocytes and in OA chondrocytes isolated from relatively macroscopically intact OA cartilage, suggesting that IL-1β could promote healthy cartilage as well as the “healthier” parts of OA cartilage tissue to become diseased, thereby enhancing disease progression. As demonstrated here, quantitative cell morphometry was useful for identifying a specific biological fingerprint, based on trainable image segmentation and modelling. Using a CIM we showed that, in some cases, individual samples can be different from the overall cell population. Thus, it is important to characterize individual cells through a panel of shape descriptors, which would allow specific identification of single cells and examination of their response to the incubation. Overall, our approach could be used for better understanding how culture conditions, inflammation or therapeutic targeting of inflammation affect cell function and outcome, which could advance our understanding of fibroblastic and immunologically relevant characteristics of single cells and cell populations to predict cell or even tissue function.

## Data availability statement

The original contributions presented in the study are included in the article/supplementary materials, further inquiries can be directed to the corresponding author/s.

## Ethics statement

The studies involving human participants were reviewed and approved by Institutional Ethics Committee of the Albert-Ludwigs-University Freiburg. The patients/participants provided their written informed consent to participate in this study.

## Author contributions

Conceptualization, BR and MH; Data curation, BR and MH; Formal analysis, BR, MH, and MS; Investigation, SA KW, and JCL; Methodology, BR and MH; Resources, BR; Supervision, BR, MH, JL, and MS; Writing—review and editing, BR, MH, and MS. All authors contributed to the article and approved the submitted version.
